# Supplementary material for: CENTRE: a gradient boosting algorithm for Cell-type-specific ENhancer-Target pREdiction
Source: Bioinformatics. 2023 Nov 20;39(11):btad687. doi: 10.1093/bioinformatics/btad687 (PMC10666202; doi:10.1093/bioinformatics/btad687)
Supplement: btad687_Supplementary_Data [file btad687_supplementary_data.zip › Supplementary_figures_111023.docx]

CENTRE: A gradient boosting algorithm for Cell-type-specific ENhancer-Target pREdiction

Trisevgeni Rapakoulia^1#^, Sara Lopez Ruiz De Vargas^1^, Persia Akbari Omgba^1^, Verena Laupert^1$^, Igor Ulitsky^1,2,3^, Martin Vingron^1, *^

^1^Max Planck Institute for Molecular Genetics, Ihnestraße 63, 14195 Berlin, Germany, ^2^ Department of Immunology and Regenerative Biology, ^3^ Department of Molecular Neuroscience, Weizmann Institute of Science, Rehovot 76100, Israel

*To whom correspondence should be addressed

Present addresses:

^#^TR: GlaxoSmithKline plc, Gunnels Wood Rd, Stevenage SG1 2NY, United Kingdom

^$^VL: Bayer AG, Müllerstrasse 178, Berlin 13353, Germany

Supplementary Figures

|   A |
| --- |
|   B |
|   C |
|   D |

**Supplementary Figure 1:** Scatter plots (left side) representing enhancer and target signals for four ET pairs and boxplots (right side) representing target signals when the enhancer is active versus inactive. All four ET pairs interact in the GM12878 cell line according to the ChIA-PET experiment targeting RNAPII^1^. In all four examples although the Pearson correlation coeficient of enhancer-target signals is low across cell types, the target signal is higher in cell types where the enhancer is considered active resulting in significant Wilcoxon runk sum test P-value. A) Scatter plot of normalized TNFRSF1B expression and DNase signal at EH38E2786897 across 112 human cell types^2^. Green dots represent cell types with higher accessibility (upper quantile DNase signal). Boxplots of TNFRSF1B expression in cells where the EH38E2786897 is accessible (upper quantile DNase signal across 112 cells) versus cells where EH38E2786897 is not accessible. B) Scatter plot of normalized MGAT4A expression and DNase signal at EH38E2017957 across 112 human cell types^2^. Green dots represent cell types with higher accessibility (upper quantile DNase signal). Boxplots of MGAT4A expression in cells where the EH38E2017957 is accessible (upper quantile DNase signal across 112 cells) versus cells where EH38E2017957 is not accessible. C) Scatter plot of DNase signal at TENT4A gene and DNase signal at EH38E3624814 across 112 human cell types^3^. Green dots represent cell types with higher enhancer accessibility (upper quantile DNase signal). Boxplots of TENT4A DNase signal in cells where the EH38E3624814 is accessible (upper quantile DNase signal across 112 cells) versus cells where EH38E3624814 is not accessible. D) Scatter plot of RNA-seq expression for TNFRSF14-AS1gene and CRUP-EP probability at EH38E2779162 across 66 human cell types (in-house constructed dataset, http://owww.molgen.mpg.de/~CENTRE_data/In_house_contructed_datasets.zip). Green dots represent cell types with higher than 0.5 CRUP-EP probability. Boxplots of TNFRSF14-AS1gene expression in cells where the EH38E2779162 is active (>0.5 CRUP-EP) versus cells where EH38E2779162 is not active.

|   A |
| --- |
|   B |
|   C |

**Supplementary Figure 2:** F1-score of the XGBoost classifier on GM12878 RNAPII-ChIAPET^1^ ET interactions when A & B) features or category of features are excluded from the model, C) when CT-specific or Generic features are excluded from the model. CRUP -EP & PP features for target and enhancer include all the five probabilities assigned to respective bins while Regulatory Distance for enhancers and promoters include both normalized and unnormalized versions. XGBoost parameters defined as in the main manuscript: colsample_bytree=0.7, gamma=1.0, learning_rate= 0.1, max_depth=5, n_estimators=300, reg_lambda=0, subsample=0.9, scale_pos_weight=5, random_state=0.

|  |
| --- |

**Supplementary Figure 3:** Pearson Correlation Coeficient of CENTRE’s features on GM12878 RNAPII-ChIAPET^1^ dataset. CRUP -EP & PP features for enhancer and target are extracted by averaging the five probabilities assigned to the respective ET bins.

|   A |
| --- |
|   B |
|   C |
|   D |
|   E |
|   F |
|   G |
|   H |
|   I |
|   J |
|   K |
|   L |

**Supplementary Figure 4:** Distribution in positive and negative ET pairs in all BENGI datasets^4^ of: A) CRUP-EP enhaner (mean CRUP-EP probability across five enhancer bins), B) CRUP-EP gene (mean CRUP-EP probability across five promoter bins), C) CRUP-PP enhaner (mean CRUP-PP probability across five enhancer bins), D) CRUP-PP gene (mean CRUP-PP probability across five promoter bins), E) Regulatory Distance Enhancers, F) Normilized Regulatory Distance Enhancers, G) Regulatory Distance Promoters, H) Normilized Regulatory Distance Promoters, I) Genomic Distance, J) CRUP correlation, K) Combined Wilcoxon Rank sum test p-value from four datasets, L) target RNA-seq signal. Generic features’ (genomic distance, CRUP-correlation and Combined Wilcoxon Rank sum test p-value from four datasets) direction is the same across BENGI datasets, and there is significant difference between true and negative ET pairs. Target’s CT features (RNA-seq, mean CRUP-EP and mean CRUP-PP extracted from averaging the five probabilities from the respective bins) have higher signal in interacting compared to non-interacting pairs and the difference is statistically significant in all datasets. The enhancer’s CT-specific features (mean CRUP-EP and mean CRUP-PP extracted from averaging the five probabilities from the respective bins) do not show a consistent pattern across BENGI datasets, and the difference is not statistically significant. We strongly believe that this outcome relies on how BENGI negative datasets were contracted. The authors generated the negative set as follows: for each cCRE-ELS of the positive pairs they gathered all unpaired target genes whose TSS was located within the 95th percentile distance from all positive ET pairs^4^. So, noticing the same distribution for the enhancer’s features is expected. Unnormalized Regulatory Distance’s features (reg_dist_enh & reg_dist_prom) are mainly lower in interacting pairs compared to the non-interacting ones, which is expected since these features are correlated with the Genomic distance feature. Normalized Regulatory Distance’s features (norm_reg_dist_enh & norm_reg_dist_prom) have higher signal in interacting pairs compared to the non-interacting pairs since the distance element has been removed with the normalization. We must make clear though that this finding should not be confused with the finding we extract from Figures 3A & 3B in the Result section. In figures 3A & 3B the 334 pairs we gathered have the same genomic distance in the two cell lines, but lower normalized Regulatory Distance’s features in GM12878 where they interact. In the BENGI datasets interacting and non-interacting pairs do not have the same distance distribution as shown in Supplementary Figure 4I.

|   A |
| --- |
|   B |
|   C |

**Supplementary Figure 5:** Top ten features extracted from XGBoost trees for A) LCLs, B) HeLa, K562, IMR90, C) tissue eQTL and NHEC datasets, annotated by BENGI^4^. Feature Importance is defined as number of times a feature appears in XGBoost trees. The feature ranking is quite similar across cell-types and experiments with both generic and CT specific features appearing in the first 10 positions. Noteworthy, in LCLs Geuvadis dataset and Ovary eQTL datasets, the RNA-seq signal is ranked in the top position leaving behind the genomic distance, which is the most important feature in the rest datasets.

|  |
| --- |

**Supplementary Figure 6:** Top ten features extracted from XGBoost trees for the pre-trained classifier on the consensus LCL dataset. Feature ranking is based on the relative number of times a particular feature occurs in XGBoost trees (feature weight over weights of all features).

| 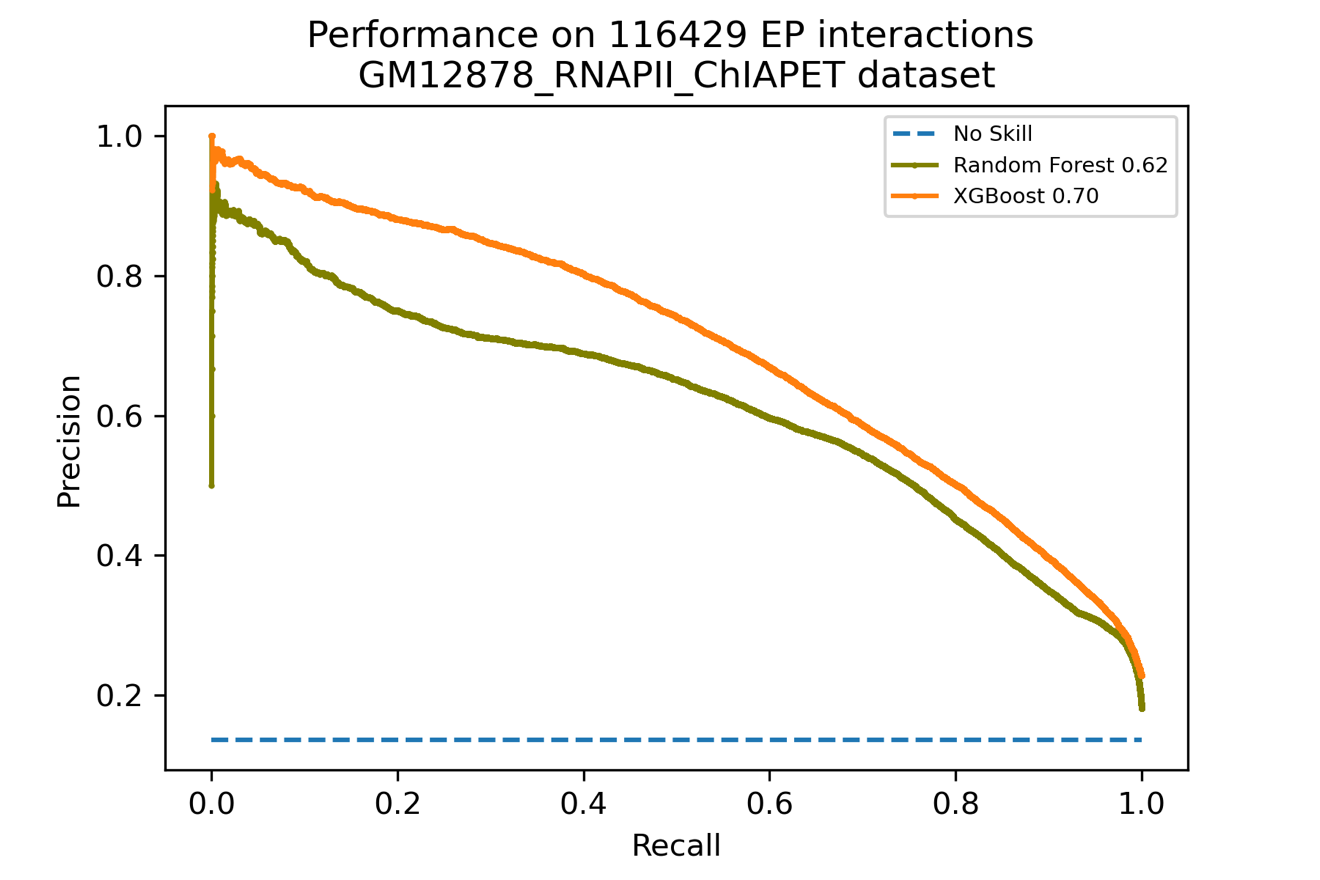  A |
| --- |
|   B |

**Supplementary Figure 7:** A) AUPRC and B) F1-score when we compare the XGBoost model with Random Forrests (RF) on GM12878 RNAPII-ChIAPET^1^ ET interactions while keeping the same feauture set between the two models. For the training of the RF classifier, we used analogous parameters with the XGBoost model. XGBoost paremeters, declared also in the main manuscript: colsample_bytree=0.7, gamma=1.0, learning_rate= 0.1, max_depth=5, n_estimators=300, reg_lambda=0, subsample=0.9, scale_pos_weight=5, random_state=0. RF parameters: n_estimators=300, class_weight = 'balanced_subsample', random_state=0, max_samples=0.9, max_depth=5.

# References

1. [Tang,Z. *et al.* (2015) CTCF-Mediated Human 3D Genome Architecture Reveals Chromatin Topology for Transcription. *Cell*, **163**, 1611–1627.](http://paperpile.com/b/mOzJ9J/g1s9V)

2.[Sheffield,N.C. *et al.* (2013) Patterns of regulatory activity across diverse human cell types predict tissue identity, transcription factor binding, and long-range interactions. *Genome Res.*, **23**, 777–788.](http://paperpile.com/b/mOzJ9J/iY721)

3. [Thurman,R.E. *et al.* (2012) The accessible chromatin landscape of the human genome. *Nature*, **489**, 75–82.](http://paperpile.com/b/mOzJ9J/ykA9T)

4. Moore,J.E. et al. (2020) A curated benchmark of enhancer-gene interactions for evaluating enhancer-target gene prediction methods. Genome Biol., 21, 17.
